# Editorial: Public health innovations for enhancing newborn and maternal wellbeing

**DOI:** 10.3389/fpubh.2026.1955843

**Published:** 2026-08-28

**Authors:** Muyiwa Oladosun, Dominic Azuh, Estelle Monique Sidze, Raymond Akawire Aborigo

**Affiliations:** 1 Covenant University, Ota, Nigeria; 2 African Population and Health Research Center (APHRC), Nairobi, Kenya; 3 Navrongo Health Research Centre, Navrongo, Ghana

**Keywords:** breastfeeding promotion intervention, health equity and crises resilience, health system strengthening, maternal and newborn care, mental health integration, newborn screening (NBS) programs

Maternal and newborn health remains a global priority, challenged by regional and in-country disparities in access, quality of care, mental health burdens, environmental risks, and system disruptions (1). Innovations in education, technology, screening, and equity-focused strategies offer promising pathways toward achieving SDG 3.2. This editorial brings together diverse studies that, are harmonized across key domains namely; post-discharge and home-based care, breastfeeding promotion and support, mental health integration, screening and early detection, system strengthening, equity in health, and crisis resilience. These contributions not only highlight unique empirical findings but also underscore the need for integrated, scalable, and context-sensitive interventions to improve outcomes for mothers and newborns worldwide (2).

## Post-discharge and home-based care

The immediate postpartum period, especially following cesarean delivery, is critical for recovery and complication prevention. Mdoe et al. developed a structured post-cesarean section home care guide and evaluated its impact through a quasi-experimental study involving 126 nurse midwives in maternity units of Dodoma and Singida regional hospitals in central Tanzania. The study findings showed education intervention was significantly associated with improved knowledge of home care after discharge (3). The guide standardizes education on key areas such as wound care, nutrition, hygiene, exercise, family planning, and mental wellbeing; directly supporting safer recoveries amid high cesarean rates in low-resource settings. This provider-focused approach ensures consistent messaging and empowers midwives as key educators (4). Leveraging on this, Ojee et al. estimated costs of implementing postnatal home-visit programs in a Kenyan level-three facility using an ingredients-based costing method aligned with the Ministry of Health recommendations. Their work delivered a practical costing tool and generalizable formula for policymakers, accounting for facility characteristics and staffing that are likely essential for scaling within Kenya's Universal Health Coverage framework (5). Thus, both studies emphasize bridging hospital-to-home gaps through practical tools and economic planning, reducing readmissions, and promoting continuity of care.

## Breastfeeding promotion and support

Optimal breastfeeding is a cornerstone of newborn nutrition and maternal health, yet barriers persist globally (6). Hu et al. synthesized evidence via systematic review and meta-analysis on remote breastfeeding guidance using telephones, text messages, and mobile applications (7). Their findings revealed significant improvements in breastfeeding rates and neonatal physical development, with stronger effects in less developed regions where access to in-person support is limited. This underscores the scalability of digital interventions for overcoming geographic and logistical challenges (8). Furthermore, cultural and systemic factors shape breastfeeding practices. Celebier et al. examined breastfeeding in Turkey, identifying common global barriers like perceived insufficient milk supply and economic constraints, alongside country-specific influences such as negative extended family dynamics. Koçakoglu advocated integrating family planning centers with breastfeeding support centers, particularly in high-fertility regions, through mobile outreach and counseling tailored to educational and sociocultural contexts. Alnimer et al. assessed healthcare professionals' knowledge, attitudes, and practices toward Baby-Friendly Hospital Initiative (BFHI) standards in Jordan. Accredited facilities showed superior performance linked to training, policies, gender, and personal breastfeeding experience (9). The four studies collectively advocate for multifaceted strategies including remote tools, cultural adaptation, policy integration, and comprehensive training to align practices with international goals.

## Mental health integration

Another dimension of maternal and newborn health is mental health integration. Maternal and paternal mental health profoundly intersects with caregiving behaviors and infant development (10). Tan Y. et al. employed quantitative methods grounded in the ABC model of behavior to examine pathways linking parental postpartum depression (PPD) to exclusive breastfeeding (EBF) intention and practice. Key insights include a notable gap between willingness and actual EBF practice, with both maternal and paternal depression exerting direct and indirect effects via family-level psychological mechanisms. Early identification and intervention emerged as critical to ameliorating maternal and paternal depression and consequences for the family. Other studies on this subject showed that complementary approaches enhance resilience (11), and that implementing person-centered approaches during the prenatal period could address postnatal depression (12). Zhu J. et al. demonstrated in a retrospective cohort that combining meditation, relaxation techniques, and prenatal multimedia health education significantly reduced fear of childbirth (tokophobia), boosted confidence, and mitigated postpartum depression without increasing adverse outcomes (13). Kalok et al. validated the Malaysian Fear of Childbirth (MyFOB) questionnaire as a reliable antenatal screening tool. Wang Z. et al. reviewed aromatherapy's efficacy for anxiety and sleep quality across pregnancy and postpartum phases. AlSaad et al. applied tabular machine learning to 410 mother-infant dyads, identifying predictors like gestational age, Edinburgh Postnatal Depression Scale/Hospital Anxiety and Depression Scale (EPDS/HADS) scores, and birth trauma for infant negative emotionality highlighted opportunities for personalized, timely interventions. These articles affirm integrating mental health support throughout the perinatal continuum of care (14).

## Early detection and treatment

Timely screening, and standardized examinations prevent long-term morbidity. Raspa et al. qualitatively assessed the U.S. newborn screening program, noting strong traditions in screening, confirmatory testing, and treatment access, with gaps existing in health education, technical assistance, follow-up, and health equity (15). The authors recommended sustained federal funding to expand capacity (15). Alemu et al. identified inconsistent neonatal examination practices and documentation in Addis Ababa hospitals, proposing national checklists and training programs for clinicians and non-physician staff to improve early anomaly detection (16). Wang S. et al. analyzed risk factors for neonatal catheter-related bloodstream infections (CRBSI), and proposed evidence-based bundles targeting dwell time, aseptic techniques, and nutrition. Eason et al. analyzed 27,184 PROM-related infant deaths (1999–2023), and documented overall declines but persistent racial (higher for Black/African American infants) and geographic disparities. Tan S. et al. reported global reductions in neonatal encephalopathy burden (1990–2021) with enduring inequities, while Zhu M. et al. projected preterm disease burdens in China, stressing modifiable risks like low birth weight and the need for strengthened rehabilitation services. Early timely screening, and standardized examinations are key evidence from these articles supporting long-term prevention of morbidity (5).

## Equity and crisis resilience

Health system strengthening, equity, and crisis resilience are correlated nexus of structural determinants shaping maternal and neonatal care. Dahir et al. used multilevel spatial analysis in Somaliland to show facility delivery strongly predicts essential newborn care receipt, with wealth quintile variations revealing unmet needs. Gadapani Pathak et al. employed mixed-methods to address anemia management barriers in rural India, recommending supply improvements, counseling, and family engagement for better compliance. Shah et al. linked social determinants and lifestyles to infant mortality in Pakistan, noting counterintuitive rural-urban differences (17). Bulut et al. revealed women's second-hand smoke (SHS) protective efforts hindered by patriarchal norms and inadequate counseling, calling for stronger healthcare roles and domestic policies. The crises contexts component of structural determinants calls for adaptability (2). Carbajal et al. synthesized COVID-19 impacts on maternity services, identifying themes in care-seeking, virtual care, vaccination, and ethics, urging women-centered, accessible strategies for future emergencies. Anikamadu et al. reviewed male involvement interventions, noting potential benefits but scalability challenges due to limited reporting on fidelity and costs. Wang H. et al. clarified delivery mode outcomes in very preterm infants via propensity matching. Collectively, these articles emphasize targeted, equity-driven system investments in maternal and newborn health care and quality.

## Conclusion

Overall, the synthesized evidence from this edition demonstrates that public health innovations spanning practical home guides, remote and digital support, mental health integration, robust screening, and equitable system strengthening can meaningfully enhance maternal and newborn wellbeing. Implementation success requires addressing cultural, socioeconomic, and logistical barriers through multidisciplinary collaboration, sustained funding, and rigorous evaluation. By prioritizing scalable, inclusive models and learning from crises, stakeholders can advance resilient quality care systems aligned with global goals like SDG 3.2 (18). Future research should focus on long-term impacts, cost-effectiveness, and replicability across diverse contexts to ensure no mother or newborn is left behind (1).
